# Supplementary material for: Target Identification and Mechanistic Characterization of Indole Terpenoid Mimics: Proper Spindle Microtubule Assembly Is Essential for Cdh1‐Mediated Proteolysis of CENP‐A
Source: Adv Sci (Weinh). 2024 Jun 14;11(29):2305593. doi: 10.1002/advs.202305593 (PMC11304278; doi:10.1002/advs.202305593)
Supplement: Supplementary file 1 — Supporting Information [file ADVS-11-2305593-s001.pdf]

ADVANCED SCIENCE
Open Access

Supporting Information

for *Adv. Sci.*, DOI 10.1002/advs.202305593

Target Identification and Mechanistic Characterization of Indole Terpenoid Mimics: Proper Spindle Microtubule Assembly Is Essential for Cdh1-Mediated Proteolysis of CENP-A

Yan Peng, Yumeng Zhang, Ruan Fang, Hao Jiang, Gongcai Lan, Zhou Xu, Yajie Liu, Zhaoyang Nie, Lu Ren, Fengcan Wang, Shou-De Zhang, Yuyong Ma, Peng Yang, Hong-Hua Ge, Wei-Dong Zhang, Cheng Luo*, Ang Li* and Weiwei He**

Target Identification and Mechanistic Characterization of Indole Terpenoid Mimics: Proper Spindle Microtubule Assembly Is Essential for Cdh1-Mediated Proteolysis of CENP-A

Yan Peng,[1,†] Yumeng Zhang,[1,†] Ruan Fang,[1,2,†] Hao Jiang,[3,†] Gongcai Lan,[1] Zhou Xu,[2] Yajie Liu,[1] Zhaoyang Nie,[2,4] Lu Ren,[2] Fengcan Wang,[1] Shou-De Zhang,[5] Yuyong Ma,[2] Peng Yang,[2,4] Hong-Hua Ge,[6] Wei-Dong Zhang,[1,7,*] Cheng Luo,[3,*] Ang Li,[2,4,*] and Weiwei He[1,*]

[1]Shanghai Key Laboratory of New Drug Design, School of Pharmacy, East China University of Science and Technology, Shanghai 200237, China

[2]State Key Laboratory of Chemical Biology, Shanghai Institute of Organic Chemistry, University of Chinese Academy of Sciences, Chinese Academy of Sciences, Shanghai 200032, China

[3]Drug Discovery and Design Center, State Key Laboratory of Drug Research, Shanghai Institute of Materia Medica, Chinese Academy of Sciences, Shanghai 201203, China

[4]Henan Institute of Advanced Technology and College of Chemistry, Zhengzhou University, Zhengzhou 450001, China

[5]State Key Laboratory of Plateau Ecology and Agriculture, Qinghai University, Xining 810016, China

[6]Institute of Physical Science and Information Technology, Anhui University, Hefei 230601, China

[7]Department of Phytochemistry, School of Pharmacy, Second Military Medical University, Shanghai 200433, China

[†]These authors contributed equally.

*Correspondence: wdzhangy@hotmail.com; cluo@simm.ac.cn; ali@sioc.ac.cn; heweiwei@ecust.edu.cn

I Materials and Methods

II Figures and Table

III References

I Materials and Methods

Preparation of JP18 analogs. JP18 analogs were prepared in racemic form by using our previously developed conjugate addition approach.[1–3]

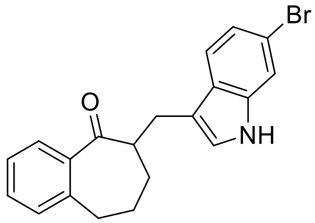

8: 6-Br-JP18

6-Br-JP18 (8): White foam. R_f = 0.23 (silica gel, EtOAc:petroleum ether 1:7); IR (film): ν_{max} = 3422, 2933, 2859, 1734, 1672, 1597, 1453, 1334, 1276, 1254, 1048, 802, 737 cm^{-1}; ^{1}H NMR (400 MHz, $CDCl_3$): δ = 7.99 (br s, 1 H), 7.62 (d, *J* = 7.5 Hz, 1H), 7.51–7.42 (m, 2 H), 7.40–7.32 (m, 1 H), 7.25 (t, *J* = 7.4 Hz, 1 H), 7.22–7.13 (m, 2 H), 6.95 (s, 1 H), 3.37 (dd, *J* = 14.5, 6.4 Hz, 1 H), 3.29–3.14 (m, 1 H), 3.04–2.84 (m, 3 H), 2.10–1.91 (m, 2 H), 1.76–1.54 (m, 2 H) ppm; ^{13}C NMR (101 MHz, $CDCl_3$): δ = 207.4, 142.2, 140.0, 137.0, 131.4, 129.9, 128.2, 126.5, 126.4, 123.0, 122.5, 120.1, 115.4, 114.5, 114.0, 50.7, 33.6, 30.3, 26.3, 25.5 ppm; HRMS (*m/z*): calcd for $C_{20}H_{18}BrNONa^+$ ([M + Na]$^+$): 390.0464, found: 390.0459.

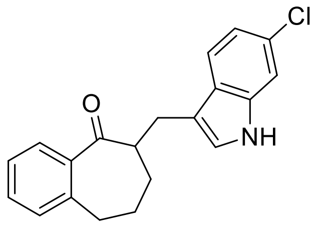

9: 6-Cl-JP18

6-Cl-JP18 (9): White foam. R_f = 0.33 (silica gel, EtOAc:petroleum ether 1:4); IR (film): ν_{max} = 3424, 2935, 2859, 1678, 1619, 1597, 1455, 1336, 804, 737 cm^{-1}; ^{1}H NMR (400 MHz, $CDCl_3$): δ = 7.96 (br s, 1 H), 7.61 (d, *J* = 7.7 Hz, 1 H), 7.49 (d, *J* = 8.5 Hz, 1 H), 7.40–7.32 (m, 1 H), 7.32 (d, *J* = 1.6 Hz, 1 H), 7.28–7.22 (m, 1 H), 7.19 (d, *J* = 7.5 Hz, 1 H), 7.06 (dd, *J* = 8.5, 1.7 Hz, 1 H), 6.97 (d, *J* = 1.9 Hz, 1 H), 3.38 (dd, *J* = 14.6, 6.4 Hz, 1 H), 3.26–3.15 (m, 1 H), 3.04–2.89 (m, 2 H), 2.90 (dd, *J* = 14.6, 7.2 Hz, 1 H), 2.09–1.92 (m, 2 H), 1.75–1.59 (m, 2 H) ppm; ^{13}C NMR (101 MHz, $CDCl_3$): δ = 207.4, 142.2, 140.1, 136.6, 131.4, 130.0, 128.3, 128.0, 126.5, 126.4, 123.1, 120.1, 119.9, 114.7, 111.1, 50.8, 33.7, 30.4, 26.4, 25.6 ppm; HRMS (*m/z*): calcd for $C_{20}H_{18}ClNONa^+$ ([M + Na]$^+$): 346.0969, found: 346.0966.

Reagents. MG-132 was purchased from Selleck Chemicals. Nocodazole, vinblastine, paclitaxel, chloroquine (CQ), and cycloheximide (CHX) were purchased from MedChemExpress. NH_4Cl was purchased from Adamas-beta.

Antibodies.

**Antibody**	**Vendor**	**Catalog No.**
Rabbit anti-Cyclin B1 (D5C10) Antibody	ABclonal	A19037
Mouse anti-Cdc2 (POH1) Antibody	CST	9116
Rabbit anti-Phospho-Cdc2 (Tyr15) Antibody	CST	4539
Rabbit anti-Phospho-Histone H3 (S10) Antibody	CST	3377
Rabbit anti-Phospho-Histone H3 (S28) Antibody	ABclonal	AP0839
Rabbit anti-Histone H3 Antibody	ABclonal	A2348
Rabbit anti-Phospho-Histone H2AX (Ser139) Antibody	Abcam	ab81299
Rabbit anti-Bcl-2 Antibody	Abways	CY5032
Rabbit anti-Cleaved PARP Antibody	Bimake	A5034
Mouse anti-α-Tubulin Antibody	Sigma−Aldrich	T9026
Rabbit anti-CENP-A Antibody	CST	2186
Rabbit anti-CENP-A Antibody	Abcam	Ab45694
Rabbit anti-GAPDH Antibody	Abways	AB0037
Rabbit anti-CDKN1A/P21CIP1 Antibody	ABclonal	A21772
Rabbit anti-LC3B Antibody	Sigma−Aldrich	L7543
Mouse anti-FLAG Tag Antibody	Affinity	T0003
Mouse anti-FLAG Tag Antibody	Bimake	A5712
Rabbit anti-Cdc20 Antibody	ABclonal	A15656
Mouse anti-Cdh1 (Ab-2) Antibody	Sigma−Aldrich	CC43
Mouse anti-Myc Tag (9B11) Antibody	CST	2276
Rabbit anti-Cullin 4A Antibody	CST	2699
Rabbit anti-DCAF11 Antibody	ABclonal	A15519
Rabbit anti-APC2 Antibody	Proteintech	13559-1-AP
Peroxidase-AffiniPure Goat anti-Rabbit IgG (H+L)	Jackson ImmunoReserch	147832
Peroxidase-AffiniPure Goat anti-Mouse IgG (H+L)	Jackson ImmunoReserch	139283
Goat anti-Rabbit IgG (H+L) (Alexa Fluor 647)	Abcam	ab150079
Goat anti-Mouse IgG (H+L) (Alexa Fluor 488)	Abcam	ab150113

Cell culture. Human cells (HeLa, MDA-MB-231, A549, U251, Hep G2, and L-02) were purchased from the Cell Bank of Chinese Academy of Sciences. HeLa, Hep G2, and U251 cells were cultured in HyClone Dulbecco's Modified Eagle's Medium (DMEM) with high glucose (Cytiva) supplemented with 10% fetal bovine serum (FBS; Biological Industries) and 1% penicillin−streptomycin (Gibco, Thermos Fisher Scientific). MDA-MB-231, A549, and L-02 cells were cultured in HyClone RPMI 1640 Medium (Cytiva) supplemented with 10% FBS and 1% penicillin−streptomycin. Cells were maintained at 37 °C with 5% CO_2 in a humidified incubator.

Plasmids. Human ubiquitin, SUMO1, and NEDD8 were cloned into the mammalian expression vector pcDNA3.1(+) (Thermo Fisher Scientific). The FLAG sequence was inserted at the N-terminus of ubiquitin. The Myc sequence was inserted at the N-termini of SUMO1 and NEDD8. Human Cdh1 isoform 1 (UniProt ID: Q9UM11-2) and isoform 2 (UniProt ID: Q9UM11-1) were cloned into the mammalian expression vector pCMV-N-FLAG (Addgene).

Transfection. Transfection was performed by using Lipofectamine 2000 (Thermo Fisher Scientific) for plasmids and Lipofectamine 3000 (Thermo Fisher Scientific) for siRNA. Cells were subjected to further experiments 36–48 h after transfection. siRNAs were purchased from GenePharma.

**siRNA**	**Sequence**
siRNA targeting a non-relevant mRNA	UUCUCCGAACGUGUCACGUTT
siRNA targeting Cdh1	No. 1: GCCAGATCGTCATCCAGAA<br>No. 2: CCAACTGGAGCGTGAACTT
siRNA targeting Cdc20	No. 1: AACGGCAGGACUCCGGGCCGATT<br>No. 2: AAUGGCCAGUGGUGGUAAUGATT
siRNA targeting APC2	No. 1: ACAUAGUGUGGACUUCUUCUC<br>No. 2: AUAUAGACUCUCAAGAAGCAC
siRNA targeting DCAF11	No. 1: CCGGGAGCUGGAAUUCAAU<br>No. 2: GCUACUCUCAGAAGGCUUU

Cell viability assay. HeLa cells were seeded in a 96-well plate at a density of 1×10^4 cells per well and treated with the test compound at concentrations ranging from 0 to 10 μM for 24 h. Dimethyl sulfoxide (DMSO; Sigma−Aldrich) was used as the vehicle control for the compound. Cell viability was determined by using the Cell Counting Kit-8 (CCK-8; Dojindo Molecular Technologies).

Cell cycle analysis. HeLa cells were seeded in a 6-well plate at a density of 1×10^6 cells per well and treated with the test compound at the indicated concentrations for 8 h. DMSO was used as the vehicle control for the compound. The cells were stained by using the Cell Cycle and Apoptosis Analysis Kit (Beyotime). Cellular DNA content was measured with a CytoFLEX S flow cytometer (Beckman Coulter), and the data obtained were analyzed with FlowJo 7.6.

Immunoblot analysis. Cells were lysed in RIPA Buffer (Cell Signaling Technology) supplemented with Protease Inhibitor Cocktail (Roche), Phosphatase Inhibitor Cocktail (Bimake), and phenylmethanesulfonyl fluoride (PMSF; Beyotime). Proteins from the cell lysate were separated by sodium dodecyl sulfate−polyacrylamide gel electrophoresis (SDS−PAGE) and transferred to a polyvinylidene difluoride (PVDF) membrane (Sigma−Aldrich). The PVDF membrane was blocked with 5% skim milk in Tris-buffered saline containing 0.1% Tween 20 (TBST) and incubated with primary and secondary antibodies. The membrane was then treated with Tanon High-sig ECL Western Blotting Substrate (Tanon), and the immunoreactive bands on the membrane were visualized by using the Tanon 5200 Chemiluminescent Imaging System (Tanon). Quantitative analysis of the immunoblot data was performed with Image J.

Immunofluorescent analysis. HeLa cells were cultured on glass-bottom culture dishes (NEST). The cells were fixed with 4% paraformaldehyde for 10 min and then permeabilized with phosphate buffered saline (PBS) containing 10% FBS and 0.5% saponin for 10 min. Samples were incubated with primary and secondary antibodies and then mounted by using Fluoroshield with DAPI (Sigma−Aldrich). Fluorescence images were captured with a Leica Stellaris 5 SR laser confocal microscope and analyzed with Leica Application Suite X (LAS X).

DARTS-based target identification. DARTS-based target identification was conducted by using Huang's protocol.[4,5] HeLa cells were lysed with M-PER Mammalian Protein Extraction Reagent (Thermo Fisher Scientific). The lysate was divided into identical aliquots and treated with compound (+)-**8** at various concentrations or the vehicle control (DMSO) at room temperature for 1 h. The samples were then incubated with thermolysin (Sigma−Aldrich) at various dilutions ranging from 1:5 to 1:160 at room temperature for 30 min. The protein mixtures were separated by SDS−PAGE and stained with Coomassie Brilliant Blue (Maokang Biotechnology). The protein bands were analyzed by mass spectrometry at Institute of Biomedical Science (IBS), Fudan University.

Tubulin polymerization assay. The tubulin polymerization assay was performed by using the HTS-Tubulin Polymerization Assay Biochem Kit (Cytoskeleton).

Protein expression and purification. The RB3 protein stathmin-like domain (RB3-SLD) and tubulin tyrosine ligase (TTL) were obtained as reported.[6] RB3-SLD was cloned into the pET22b vector and overexpressed in *Escherichia coli*. The protein was purified by anion-exchange chromatography on a Q Sepharose Fast Flow column (GE Healthcare) and fast protein liquid chromatography on a Superose 12 HR 10/30 gel filtration column (Amersham Pharmacia Biotech) and then concentrated to 10 mg/mL. TTL was cloned into the pET22b vector with a hexahistidine tag. The protein was purified by immobilized metal affinity chromatography on a HisTrap HP column (GE Healthcare) and size exclusion chromatography on a Superdex 200 column (GE Healthcare) and then concentrated to 10 mg/mL.

Crystallization and crystal soaking. The T2R−TTL complex was formed by mixing tubulin (bovine brain; Cytoskeleton), RB3-SLD, and TTL in a molar ratio of 2:1.3:1.2.[6] The protein complex was concentrated to 20 mg/mL before adding 1 mM AMP-PCP, 5 mM tyrosinol, and 10 mM dithiothreitol (DTT). Crystallization was performed by using the sitting-drop vapor diffusion method at 16 °C. Crystals grew in well solutions at pH 6.5–6.9 containing 6–10% PEG 4000, 4–10% glycerol, 30 mM $MgCl_2$, and 100 mM MES/imidazole, reaching stable dimensions within 4 d. The resulting crystals were soaked in a well solution containing the test compound (1 mM) for 12–24 h.

Data collection and structure determination. The soaked crystals were cryoprotected by snap-freezing in liquid nitrogen with a well solution containing ethylene glycol (20%) and the test compound (1 mM). Diffraction data were collected on beamlines BL17U1 and BL19U1 at Shanghai Synchrotron Radiation Facility (SSRF). The data were processed with HKL3000.[7] The structure of the T2R−TTL−MTA complex was determined by molecular replacement with the T2R−TTL structure (PDB ID: 4O2B) as a search model in Phenix.[8] The model for T2R−TTL−MTA was built in COOT[9] and refined in Phenix.[8] Coordinates were deposited in the Protein Data Bank (PDB) with identification codes 7CPD [T2R−TTL−(+)-**8**] and 7CPQ [T2R−TTL−(+)-**9**]. For comprehensive statistics on data collection and model refinement, refer to Table S1.

RNA extraction, reverse transcription, and qRT-PCR. HeLa cells were seeded in a 6-well culture plate at a density of 4×10^5 cells per well and treated with (+)-**8** (0.5 μM), (+)-**9** (0.5 μM), nocodazole (0.1 μM), and vinblastine (0.05 μM) for 6 h. DMSO was used as the vehicle control for the compound. Total RNA was extracted with TRIzol Reagent (Invitrogen). Reverse transcription was performed by using the PrimeScript RT Reagent Kit with gDNA Eraser (Takara). qRT-PCR was performed by using the QuantStudio Real-Time PCR System (Thermo Fisher Scientific). The β-actin mRNA level was used as an internal reference for normalization. The following sense (S) and antisense (AS) primers were used for qRT-PCR amplification.

CENP-A (S): 5′-CTTCCTCCCATCAACACAGTCG-3′.

CENP-A (AS): 5′-TGCTTCTGCTGCCTCTTGTAGG-3′.

β-actin (S): 5′-GAGCGGGAAATCGTGCGTGACATT-3′.

β-actin (AS): 5′-GATGGAGTTGAAGGTAGTTTCGTG-3′.

Statistical analysis. Statistical analysis was performed by using GraphPad Prism 5.0. A unpaired two-tailed Student's *t*-test was used to compare two groups. A one-way analysis of variance (ANOVA) followed by Tukey's multiple comparison test was used to compare multiple groups with a single control. Differences were considered statistically significant if $P < 0.05$.

II Figures and Table

Figure S1. Cell-cycle-arresting effect of JP18 (**1**) and its analogs (**2**–**16**). HeLa cells were treated with the indicated compounds at the specified concentrations for 8 h and then stained with PI. Cell cycle distribution was analyzed by using flow cytometry. DMSO was used as the vehicle control for the compounds.

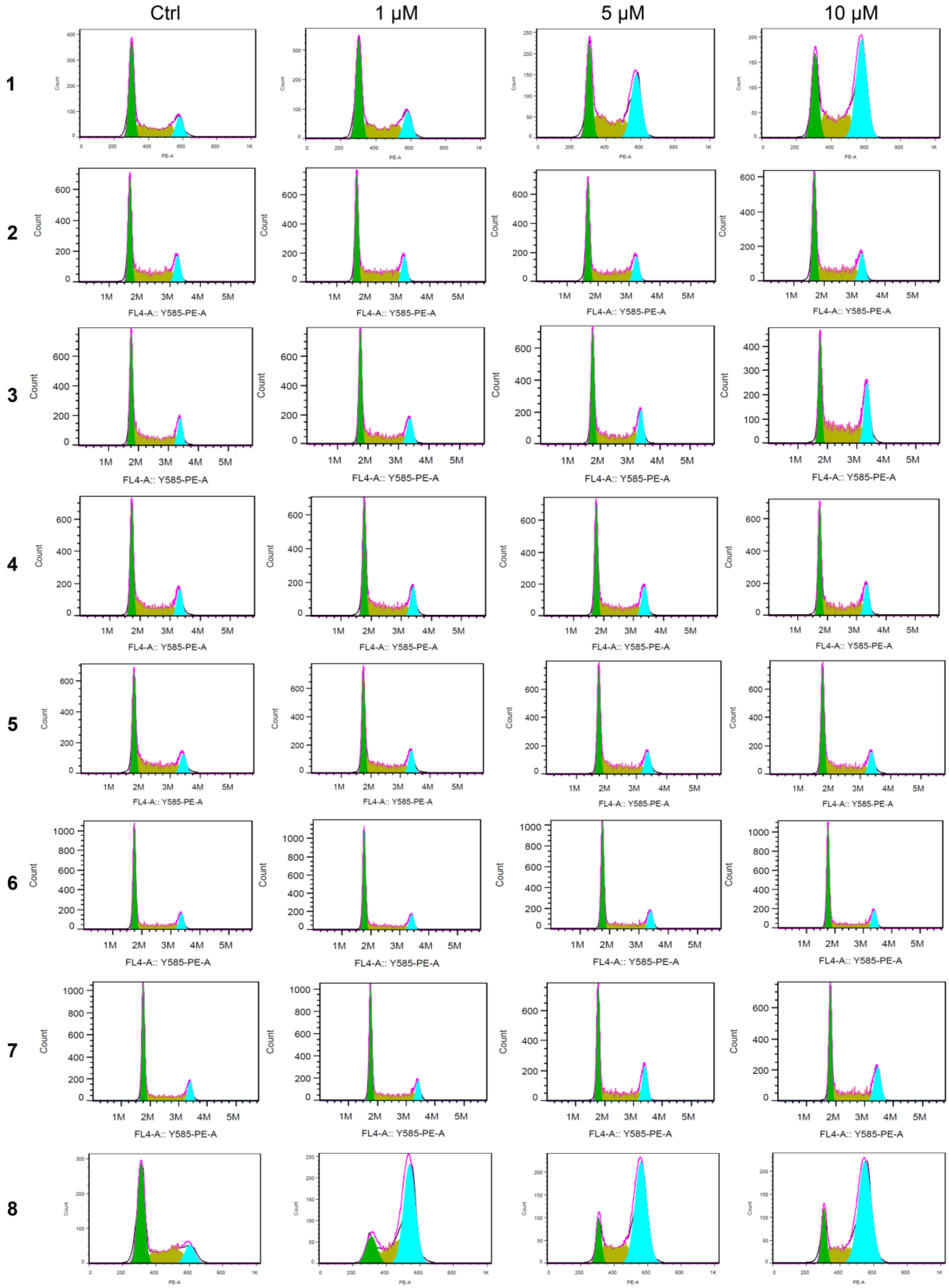

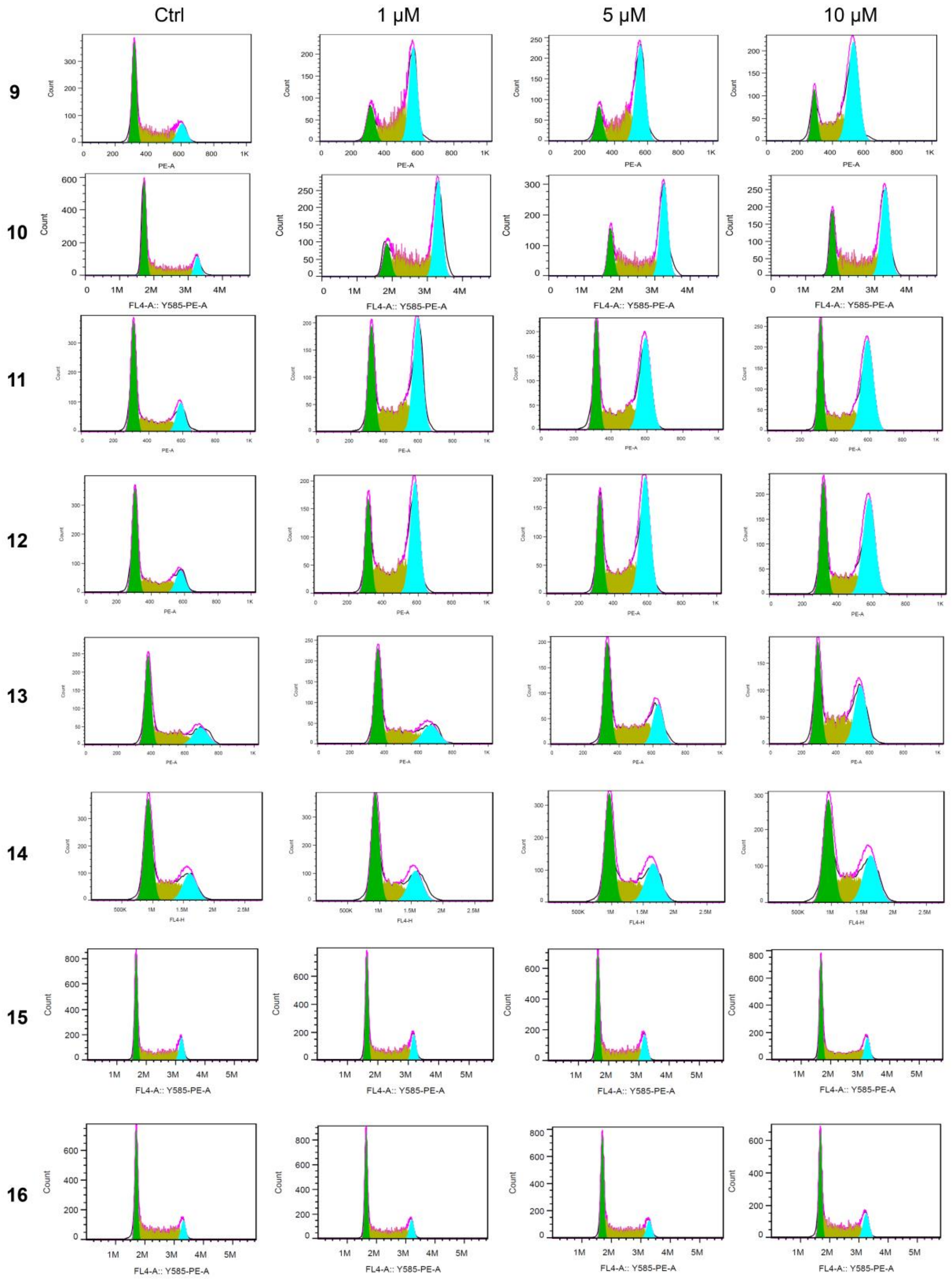
Ctrl
1 μM
5 μM
10 μM
9
10
11
12
13
14
15
16
Count
PE-A
FL4-A:: Y585-PE-A
FL4-H

Figure S2. Compounds **8** and **9** exhibit stronger G2/M-phase-arresting activity compared to compound **1**. A) Compounds **8** and **9** induced cell cycle arrest in the G2/M phase in a dose-dependent manner, even at low concentrations. HeLa cells were treated with compounds **1**, **8**, and **9** at the indicated concentrations for 8 h. B) Compounds **8** (0.5 µM) and **9** (0.5 µM) induced cell cycle arrest in G2/M phase in a time-dependent manner. HeLa cells were treated with compounds **1** (0.5 µM), **8** (0.5 µM), and **9** (0.5 µM) for the indicated durations. Bar graphs depict the percentages of cells in the G2/M phase (green), S phase (yellow), and G1 phase (orange). DMSO was used as the vehicle control for the compounds.

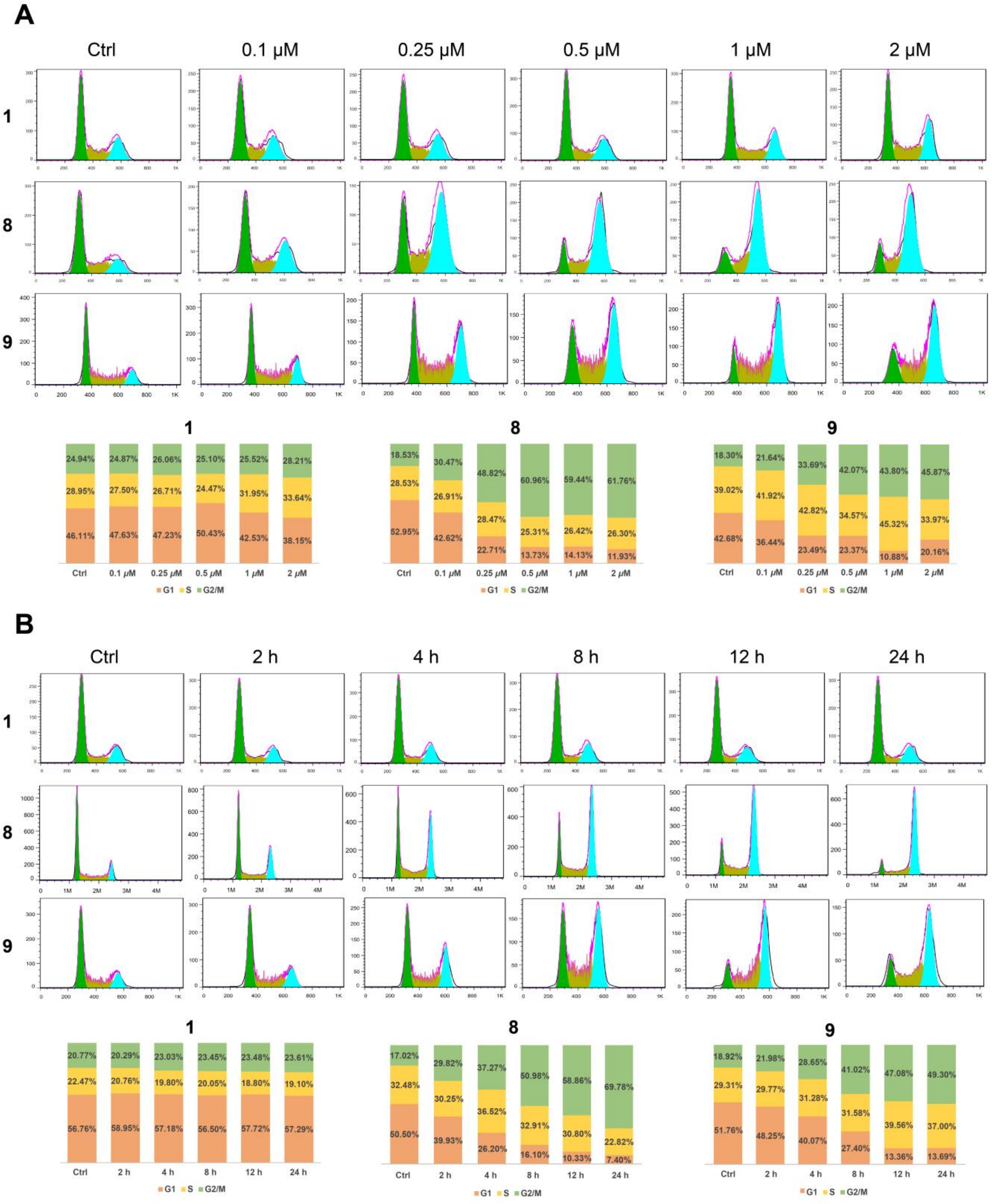

Figure S3. Dose−response curves for determining the IC_{50} values of compounds **1**, **8** and **9** in HeLa cells. HeLa cells were treated with the indicated compounds at concentrations ranging from 0 to 10 μM for 24 h. Cell viability was assessed with the CCK-8 assay. The IC_{50} values were calculated by using GraphPad Prism 5.0.

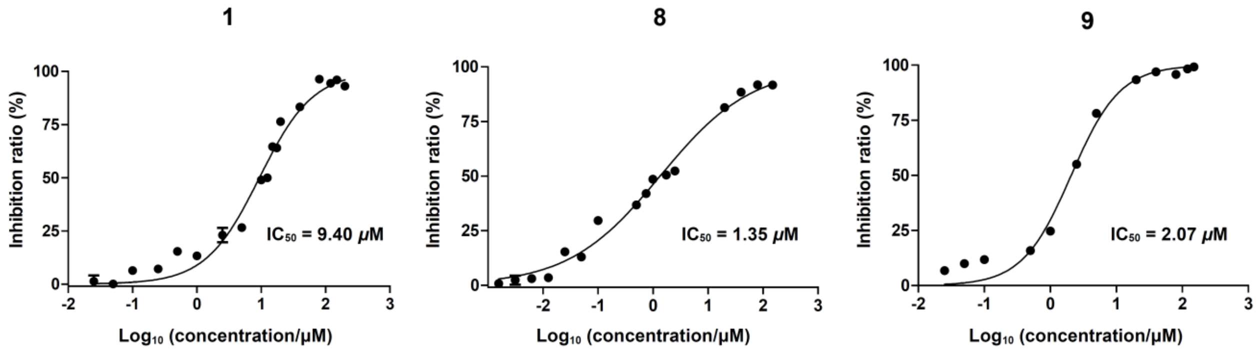

Figure S4. Cell-cycle-arresting effect of the enantiomeric forms of compounds **8** and **9**. A) The cell-cycle-arresting effect of (+)-**8** and (−)-**8**. HeLa cells were treated with the indicated compounds at the specified concentrations for 8 h. B) The cell-cycle-arresting effect of (+)-**9** and (−)-**9**. HeLa cells were treated with the indicated compounds at the specified concentrations for 8 h. DMSO was used as the vehicle control for the compounds.

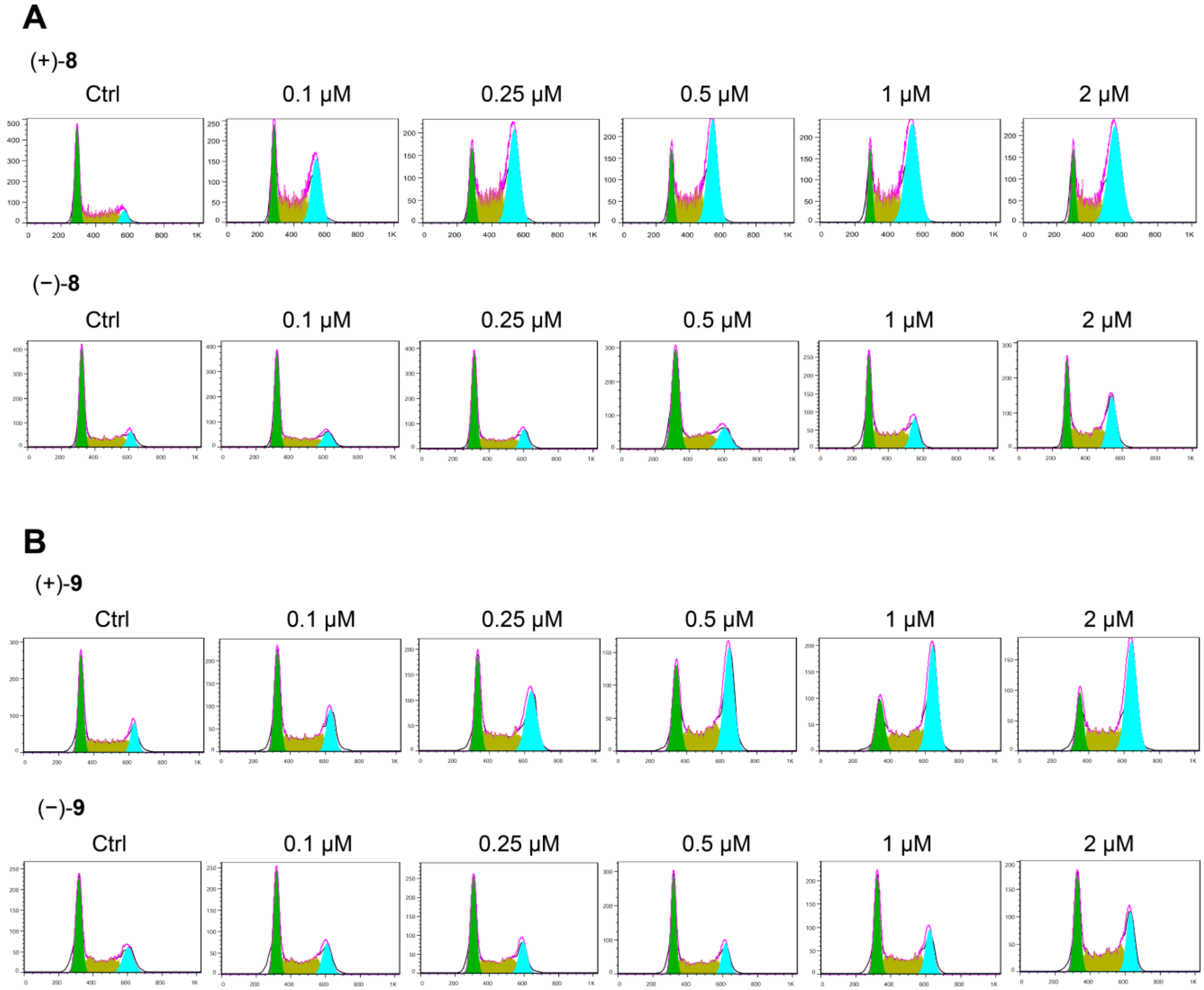

Figure S5. Effect of compounds (+)-**8** and (+)-**9** on markers of cell survival, DNA damage, and apoptosis. A,B) Immunoblot analysis of Bcl-2, γ-H2AX, and cleaved PARP1 in HeLa cells treated with (+)-**8** and (+)-**9**, respectively, at the indicated concentrations for 24 h. C,D) Immunoblot analysis of Bcl-2, γ-H2AX, and cleaved PARP1 in HeLa cells treated with (+)-**8** (0.5 μM) and (+)-**9** (0.5 μM), respectively, for the indicated durations. DMSO was used as the vehicle control for the compounds, and GAPDH was used as the loading control for immunoblotting.

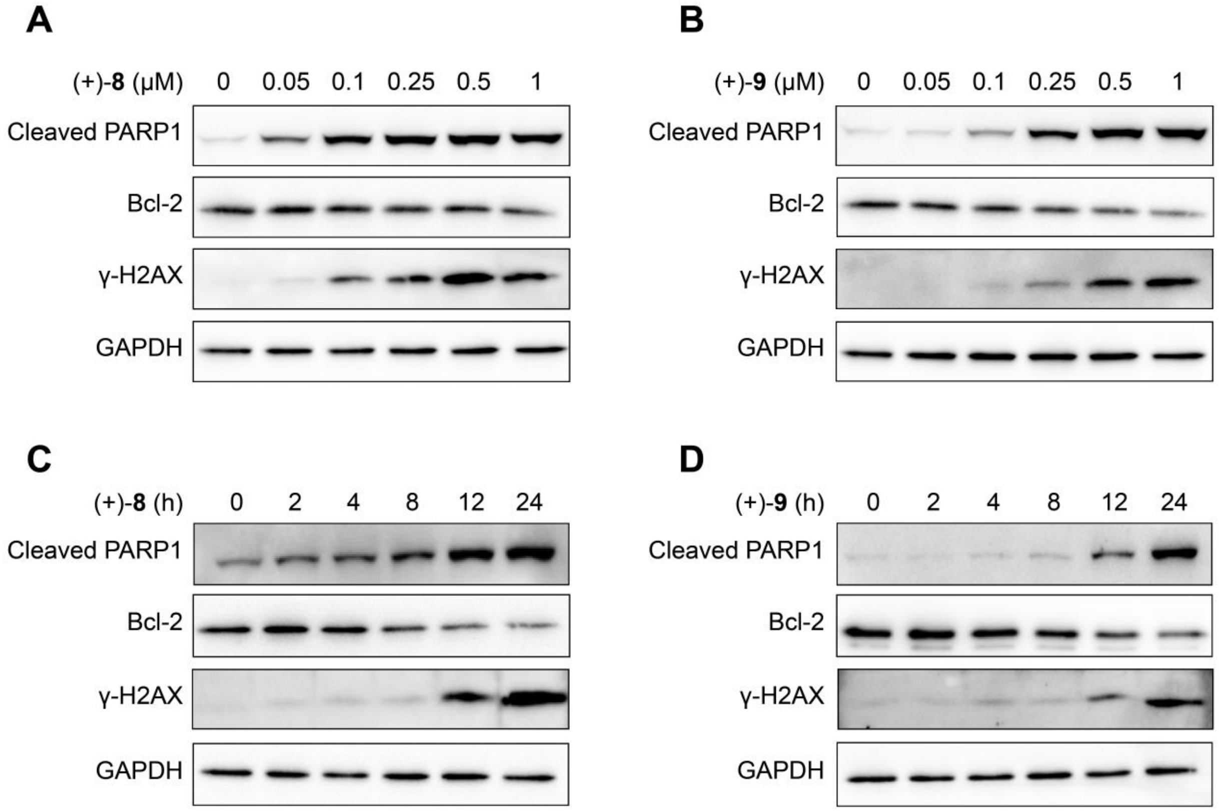

Figure S6. X-ray crystal structure of the T2R−TTL−(+)-**9** complex. α-Tubulin (light slate blue), β-tubulin (green), RB3-SLD (plum), and TTL (gold) are shown in cartoon representation, while compound (+)-**9**, GDP, and GTP are shown in sphere representation.

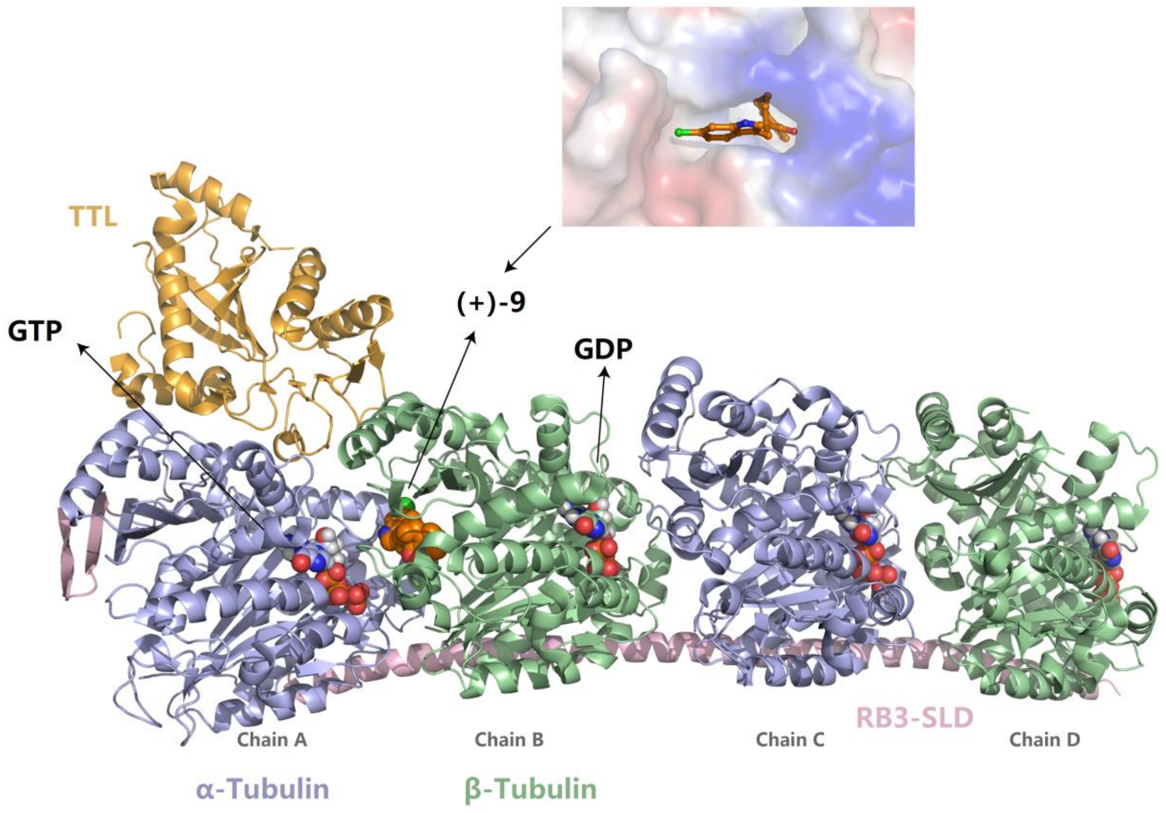

Figure S7. Characterization of the post-translational regulation of CENP-A. A) Immunoblot analysis of CENP-A in HeLa cells treated with NH_4Cl (40 mM) for 3 h. B) Immunoblot analysis of CENP-A in HeLa cells treated with CQ (30 μM) for 3 h. LC3B was used as a positive control for lysosome inhibition. C) Immunoblot analysis of CENP-A in HeLa cells transfected with Myc−NEDD8. EV = empty vector. D) Immunoblot analysis of CENP-A in HeLa cells transfected with Myc−SUMO1. E) Comparison of CENP-A levels in HeLa cells treated with nocodazole (0.1 μM) alone versus in those co-treated with nocodazole (0.1 μM) and MG-132 (50 μM) for the indicated durations. *4 h. **6 h. GAPDH was used as the loading control for immunoblotting.

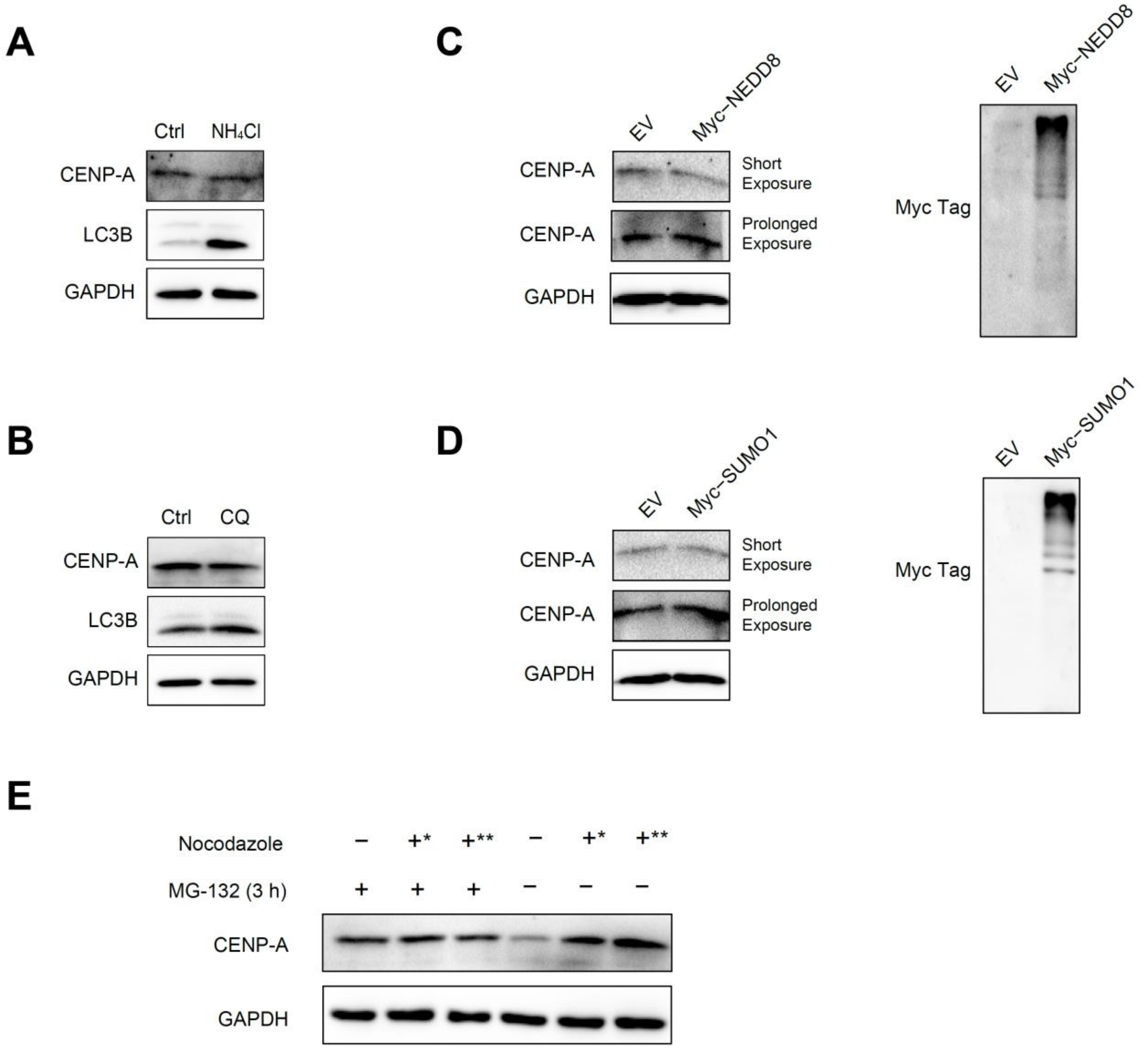

Figure S8. Compound (+)-**8** and paclitaxel downregulate Cdh1 and upregulate CENP-A in various human cells. A) Immunoblot analysis of CENP-A, Cdh1, and Cdc20 in Hep G2 cells treated with (+)-**8** (0.5 μM) for the indicated durations. B) Immunoblot analysis of CENP-A, Cdh1, and Cdc20 in A549 cells treated with (+)-**8** (0.5 μM) for the indicated durations. C) Immunoblot analysis of CENP-A, Cdh1, and Cdc20 in L-02 cells treated with (+)-**8** (0.5 μM) for the indicated durations. D) Immunoblot analysis of CENP-A, Cdh1, and Cdc20 in Hep G2 cells treated with paclitaxel (0.2 μM) for the indicated durations. E) Immunoblot analysis of CENP-A, Cdh1, and Cdc20 in A549 cells treated with paclitaxel (0.2 μM) for the indicated durations. F) Immunoblot analysis of CENP-A, Cdh1, and Cdc20 in L-02 cells treated with paclitaxel (0.2 μM) for the indicated durations. DMSO was used as the vehicle control for the compounds, and GAPDH was used as the loading control for immunoblotting.

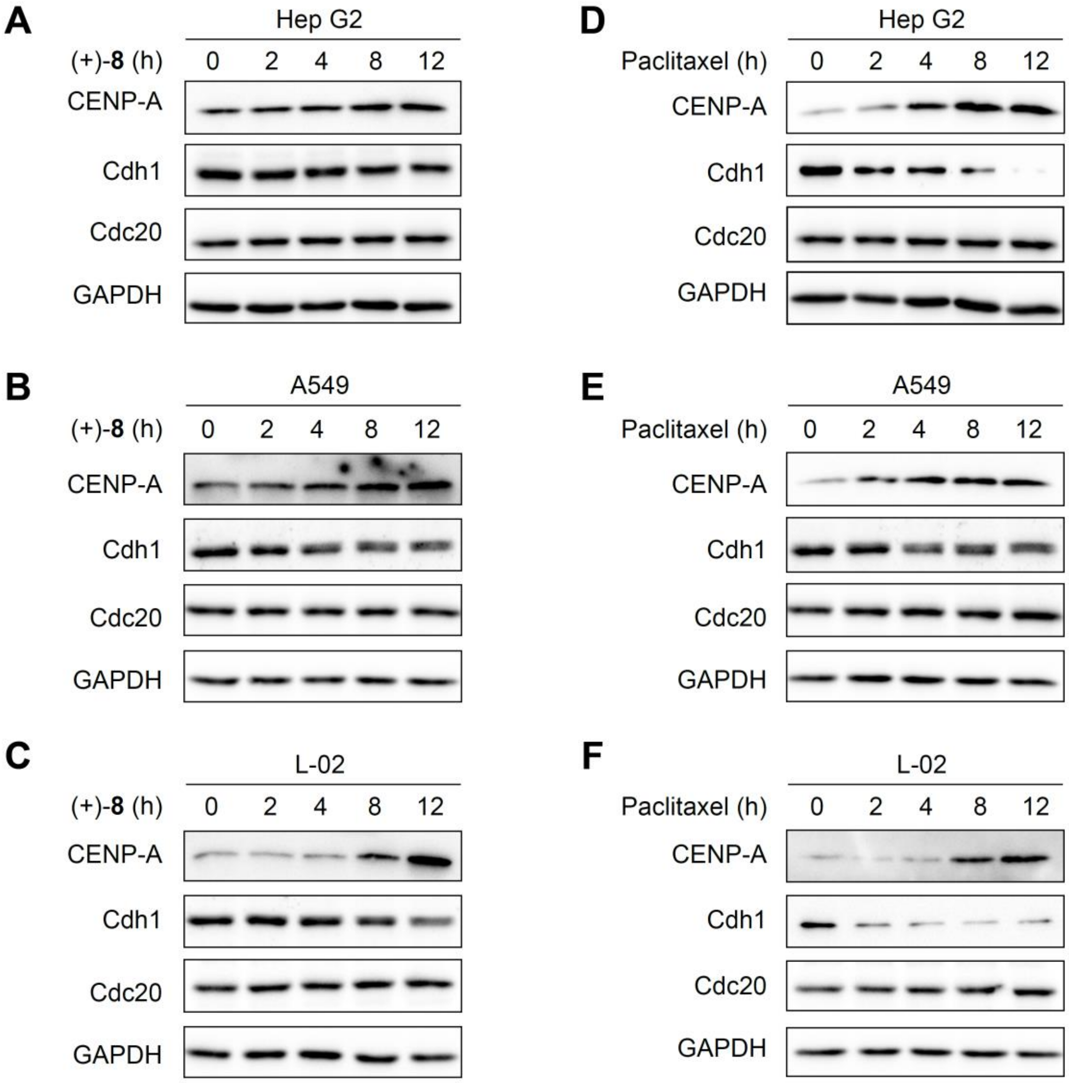

Figure S9. Compound (+)-**8** downregulates Cdh1 at the post-translational level. Cdh1 levels in HeLa cells treated with CHX (355 μM) alone versus in those co-treated with CHX (355 μM) and (+)-**8** (0.5 μM) for the indicated durations were compared by immunoblotting. DMSO was used as the vehicle control for the compound, and GAPDH was used as the loading control for immunoblotting.

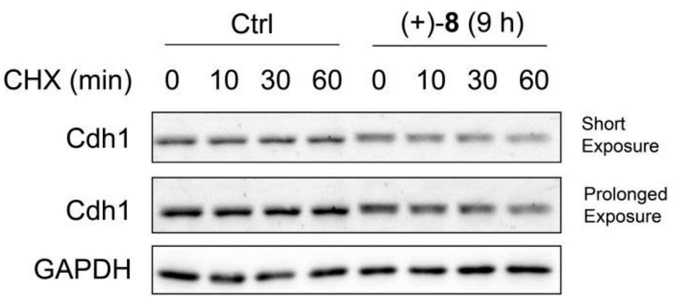

Figure S10. Cdc20 knockdown does not affect CENP-A levels. A) Immunoblot analysis of CENP-A in HeLa cells transfected with siRNA targeting Cdc20 mRNA. Two different siRNA oligos were used independently in the knockdown experiment. B) Quantitative analysis of the immunoblotting data from the above experiment. Data are presented as mean ± s.e.m. NS = not significant (significance level: α = 0.05; n = 6, two-tailed Student's t-test). siRNA targeting a non-relevant mRNA was used as the negative control for knockdown, and GAPDH was used as the loading control for immunoblotting.

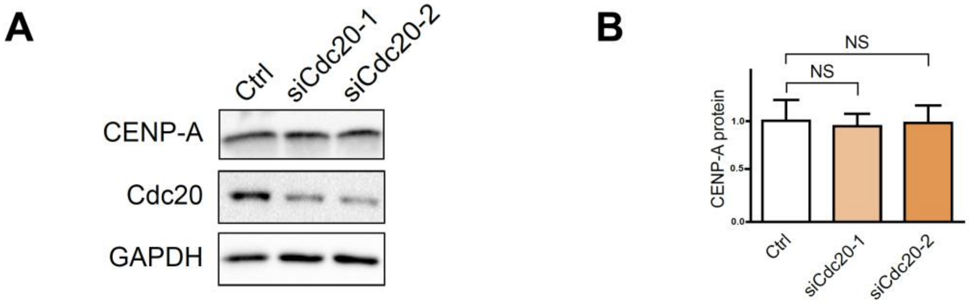

Figure S11. Cdh1 knockdown increases CENP-A levels in various human cells. A–E) Immunoblot analysis of CENP-A in Hep G2, A549, MDA-MB-231, U251, and L-02 cells transfected with siRNA targeting Cdh1 mRNA, respectively. Two different siRNA oligos were used independently in each knockdown experiment. siRNA targeting a non-relevant mRNA was used as the negative control for knockdown, and GAPDH was used as the loading control for immunoblotting.

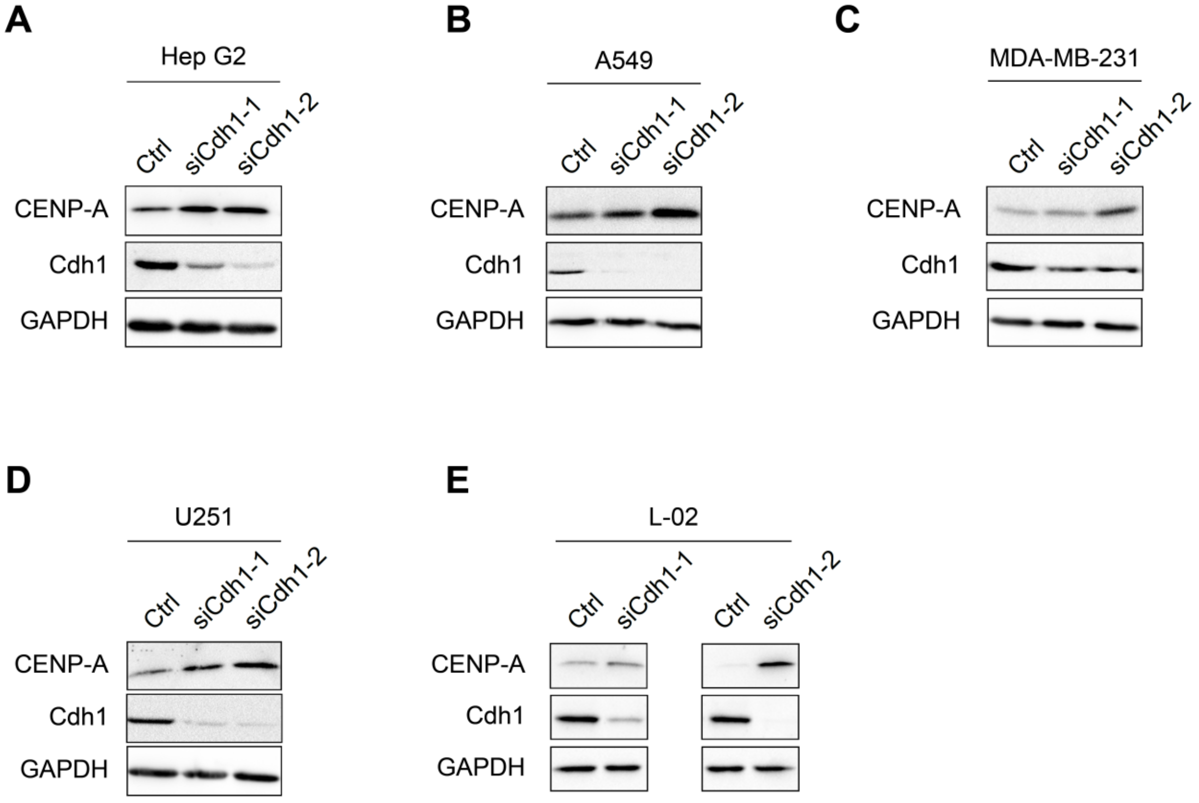

Figure S12. Combination of Cdh1 knockdown and MTA treatment enhances CENP-A accumulation compared to MTA treatment alone. A) Immunoblot analysis of CENP-A in HeLa cells transfected with siRNA targeting Cdh1 mRNA and then treated with (+)-**8** (0.5 μM) for 12 h. B) Immunoblot analysis of CENP-A in HeLa cells transfected with siRNA targeting Cdh1 mRNA and then treated with paclitaxel (0.2 μM) for 12 h. C) Immunoblot analysis of CENP-A in HeLa cells transfected with siRNA targeting DCAF11 mRNA and then treated with (+)-**8** (0.5 μM) for 12 h. D) Immunoblot analysis of CENP-A in HeLa cells transfected with siRNA targeting DCAF11 mRNA and then treated with paclitaxel (0.2 μM) for 12 h. Two different siRNA oligos were used independently in each knockdown experiment. DMSO was used as the vehicle control for the compounds; siRNA targeting a non-relevant mRNA was used as the negative control for knockdown; GAPDH was used as the loading control for immunoblotting.

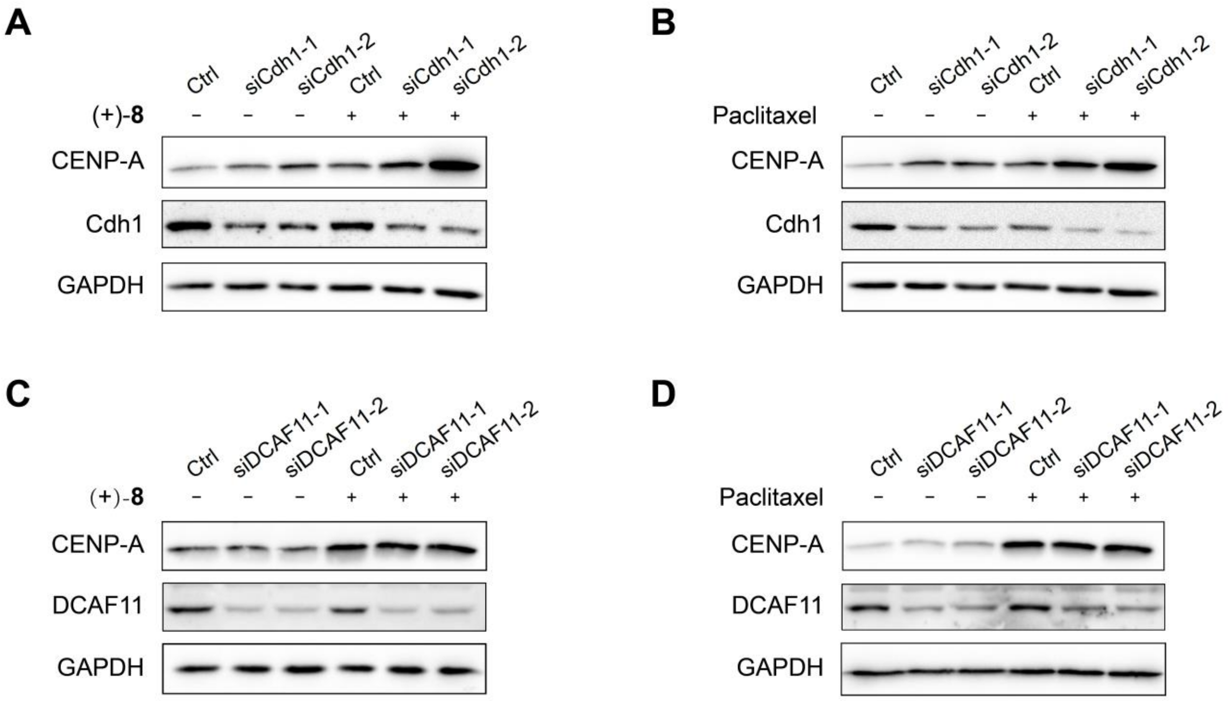

Figure S13. Combination of APC2 knockdown and MTA treatment enhances CENP-A accumulation compared to MTA treatment alone. A) Immunoblot analysis of CENP-A in HeLa cells transfected with siRNA targeting APC2 mRNA and then treated with (+)-**8** (0.5 μM) for 12 h. B) Immunoblot analysis of CENP-A in HeLa cells transfected with siRNA targeting APC2 mRNA and then treated with paclitaxel (0.2 μM) for 12 h. Two different siRNA oligos were used independently in each knockdown experiment. DMSO was used as the vehicle control for the compounds; siRNA targeting a non-relevant mRNA was used as the negative control for knockdown; GAPDH was used as the loading control for immunoblotting.

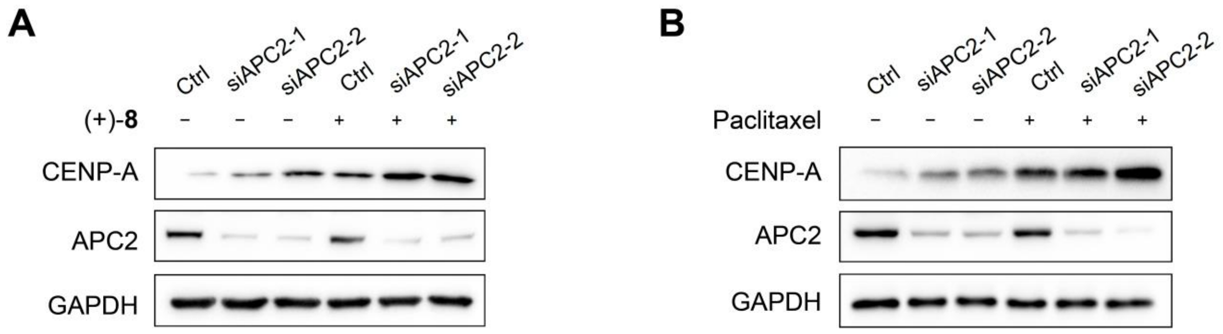

Figure S14. Compound (+)-**8** and paclitaxel do not significantly affect CUL4A or DCAF11 levels. A) Immunoblot analysis of CUL4A and DCAF11 in HeLa cells treated with (+)-**8** (0.5 μM) for the indicated durations. B) Immunoblot analysis of CUL4A and DCAF11 in HeLa cells treated with paclitaxel (0.2 μM) for the indicated durations. DMSO was used as the vehicle control for the compounds, and GAPDH was used as the loading control for immunoblotting.

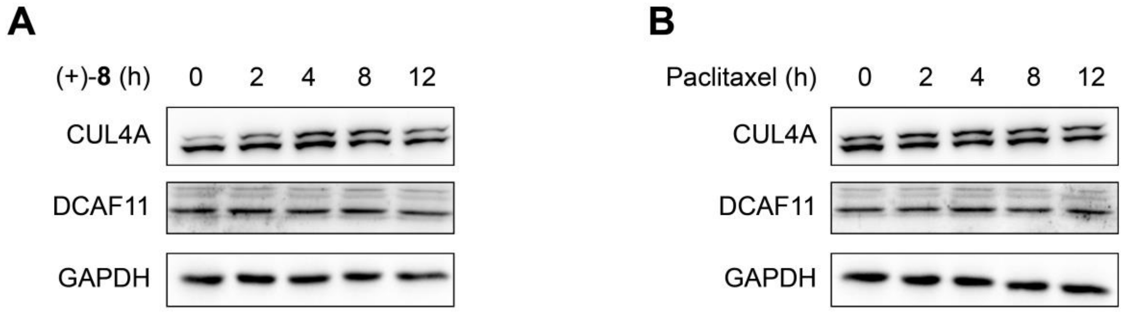

Table S1. Comprehensive statistics on data collection and model refinement.

Parameter	Data for T2R−TTL−(+)-**8**	Data for T2R−TTL−(+)-**9**
Wavelength	0.9785	0.9875
Resolution range	49.89–2.506 (2.596–2.506)	48.06–2.595 (2.688–2.595)
Space group	P 21 21 21	P 21 21 21
Unit cell dimensions	104.782, 156.552, 182.591	104.877, 156.616, 182.624
Unit cell angles	90, 90, 90	90, 90, 90
Total reflections	1,335,935 (107,124)	1,201,389 (76,058)
Unique reflections	102,800 (8,435)	92,838 (7,549)
Redundancy	13.0 (12.7)	12.9 (11.4)
Completeness (%)	99.4 (99.4)	99.4 (99.7)
Mean I/σ(I)	26.40 (2.67)	23.00 (2.00)
Wilson B-factor	35.09	40.20
R-merge	0.089 (0.987)	0.206 (2.023)
R-meas	0.093 (1.028)	0.215 (2.116)
CC1/2	0.995 (0.813)	0.987 (0.708)
Reflections used in refinement	100,497 (8,434)	90,558 (7,550)
Reflections used for R-free	5,063 (434)	4,654 (380)
R-work	0.1829 (0.2288)	0.2193 (0.2954)
R-free	0.2401 (0.3027)	0.2664 (0.3548)
Number of non-hydrogen atoms	17,898	17,602
Macromolecules	17,326	17,326
Ligands	191	190
Protein residues	2,162	2,162
RMS (bonds)	0.011	0.012
RMS (angles)	0.98	0.92
Ramachandran favored (%)	95	95
Ramachandran allowed (%)	4.3	4.6
Ramachandran outliers (%)	0.60	0.78
Rotamer outliers (%)	3.7	4.2
Clashscore	17.46	14.44
Average B-factor	51.47	46.77
Macromolecules	51.65	46.87
Ligands	45.20	40.76
Solvent	46.35	39.53
Number of TLS groups	1	1

Statistics for the highest-resolution shell are shown in parentheses.

III References

[1] X. Xiong, D. Zhang, J. Li, Y. Sun, S. Zhou, M. Yang, H. Shao, A. Li, *Chem. Asian J.* **2015**, *10*, 869.

[2] Y. Sun, P. Chen, D. Zhang, M. Baunach, C. Hertweck, A. Li, *Angew. Chem., Int. Ed.* **2014**, *53*, 9012.

[3] J. Pei, S. Zhou, F. Yang, Y. Sun, A. Li, W.-D. Zhang, W. He, *Chem. Asian J.* **2016**, *11*, 2715.

[4] B. Lomenick, R. Hao, N. Jonai, R. M. Chin, M. Aghajan, S. Warburton, J. Wang, R. P. Wu, F. Gomez, J. A. Loo, J. A. Wohlschlegel, T. M. Vondriska, J. Pelletier, H. R. Herschman, J. Clardy, C. F. Clarke, J. Huang, *Proc. Natl. Acad. Sci. U. S. A.* **2009**, *106*, 21984.

[5] M. Y. Pai, B. Lomenick, H. Hwang, R. Schiestl, W. McBride, J. A. Loo, J. Huang, *Methods Mol. Biol*. **2015**, *1263*, 287.

[6] A. E. Prota, M. M. Magiera, M. Kuijpers, K. Bargsten, D. Frey, M. Wieser, R. Jaussi, C. C. Hoogenraad, R. A. Kammerer, C. Janke, M. O. Steinmetz, *J. Cell Biol*. **2013**, *200*, 259.

[7] W. Minor, M. Cymborowski, Z. Otwinowski, M. Chruszcz, *Acta Crystallogr. D* **2006**, *62*, 859.

[8] P. D. Adams, P. V. Afonine, G. Bunkoczi, V. B. Chen, I. W. Davis, N. Echols, J. J. Headd, L. W. Hung, G. J. Kapral, R. W. Grosse-Kunstleve, A. J. McCoy, N. W. Moriarty, R. Oeffner, R. J. Read, D. C. Richardson, J. S. Richardson, T. C. Terwilliger, P. H. Zwart, *Acta Crystallogr. D* **2010**, *66*, 213.

[9] P. Emsley, B. Lohkamp, W. G. Scott, K. Cowtan, *Acta Crystallogr. D* **2010**, *66*, 486.
